# Intratumor heterogeneity score reveals immune landscape and survival stratification in colorectal cancer

**DOI:** 10.3389/fimmu.2025.1671148

**Published:** 2026-01-05

**Authors:** Zijing Wang, Liyuan Ma, Jinzhong Cao, Xi Chen, Xin Chang, Ruxue Ma, Hengyi Lv, Zixin Zhang, Hai Li, Tao Jiang

**Affiliations:** 1 First Clinical Medical College, General Hospital of Ningxia Medical University, Yinchuan, China; 2 Ningxia Key Laboratory of Stem Cell and Regenerative Medicine, Institute of Medical Sciences, General Hospital of Ningxia Medical University, Yinchuan, China; 3 Department of Ultrasonography, General Hospital of Ningxia Medical University, Yinchuan, China; 4 The First Clinical Medical College of Lanzhou University, Department of Obstetrics and Gynecology, Gansu Provincial Clinical Research Center for Gynecological Oncology, Lanzhou, Gansu, China; 5 Department of Anal-Colorectal Surgery, General Hospital of Ningxia Medical University, Yinchuan, China

**Keywords:** colorectal cancer, intratumor heterogeneity, IL20RB, prognosis, immune landscape

## Abstract

**Background:**

Intratumor heterogeneity (ITH), a critical driver of tumor evolution and immune evasion, remains inadequately characterized at the transcriptomic level in colorectal cancer (CRC), and its clinical implications are not yet fully understood.

**Methods:**

We integrated transcriptomic datasets from TCGA-COAD/READ and two independent GEO cohorts (GSE40967 and GSE87211) to develop an RNA-seq–based ITH score using the DEPTH2 algorithm and to con0struct an ITH-related gene (ITRG) prognostic model. A unified cutoff value of 0.64 was established to stratify patients into high- and low-ITH groups. Using 52 survival-associated ITRGs, we generated a nine-gene prognostic signature selected from 101 distinct combinations of feature selection techniques and modeling algorithms and validated its performance in two external datasets. The tumor microenvironment and potential responsiveness to immune checkpoint inhibitors were evaluated using ssGSEA, ESTIMATE, and TIDE algorithms. SHAP analysis, together with *in vitro* and *in vivo* experiments, was employed to identify and functionally validate key regulatory genes.

**Results:**

Patients in the high-ITH group had markedly poorer overall survival (OS) than those in the low-ITH group. The ITH score correlated strongly with aggressive clinical features, including T3/4 invasion depth, N1/2 nodal status, and AJCC stage III. The nine-gene prognostic signature demonstrated consistent predictive capability across the TCGA training cohort and both GEO validation cohorts. In TCGA, the model yielded time-dependent AUCs of 0.669, 0.664, and 0.645 for 1-, 3-, and 5-year OS, respectively, and retained its status as an independent prognostic indicator in multivariate Cox regression analysis; in GSE40967 and GSE87211, the corresponding AUCs ranged from 0.544–0.573 and 0.648–0.744. The high-risk subgroup was characterized by a stromal immune phenotype enriched with cancer-associated fibroblasts and macrophages, elevated expression of multiple immune checkpoints, increased TIDE scores, and higher tumor mutational burden. SHAP analysis identified IL20RB as the top risk-associated gene, whose knockdown significantly suppressed CRC cell proliferation, migration, invasion, and tumorigenicity *in vitro* and *in vivo*.

**Conclusion:**

This study introduces and validates a transcriptomic ITH score and a nine-gene ITRG-based prognostic model that delineate the immune landscape and enables effective survival stratification in CRC, complementing the limitations of current staging systems. Additionally, IL20RB is highlighted as a promising therapeutic target, supporting the development of personalized immuno-targeted combination therapies in CRC.

## 1 Introduction

Colorectal cancer (CRC) ranks as the third most frequently diagnosed cancer and stands as the second leading cause of cancer-related mortality on a global scale (1). According to the GLOBOCAN 2020 report, there were approximately 1.93 million newly diagnosed CRC cases and 935,000 associated deaths globally (2). Projections from the World Health Organization indicate that by 2040, these numbers will rise to 3.2 million new cases and 1.6 million deaths, reflecting the growing global burden of CRC (3). Although the TNM staging system and molecular biomarkers such as microsatellite instability-high (MSI-H) and deficient mismatch repair (dMMR) are widely used to inform decisions regarding surgical intervention, adjuvant chemotherapy, and immunotherapy, their utility remains limited (4, 5). Notably, around 85% of CRC patients have microsatellite-stable (MSS) tumors, which respond poorly to immune checkpoint inhibitors (ICIs) (6). Furthermore, considerable heterogeneity in survival outcomes persists among patients classified within the same TNM stage, highlighting the inadequacy of existing classification frameworks in addressing the requirements of precision oncology (7).

In recent years, molecular subtyping systems such as the Consensus Molecular Subtypes (CMS), CRC Intrinsic Subtypes (CRIS), tumor mutational burden (TMB), and the Immunoscore have partially improved the biological stratification of CRC (8, 9). However, these frameworks remain insufficient in capturing the complex evolutionary dynamics and immune landscape inherent to this highly heterogeneous disease (10). Intratumor heterogeneity (ITH)—defined as multiclonality at the genomic, epigenetic, and phenotypic levels within a single tumor—is now widely recognized as a key driver of tumor progression, immune evasion, and acquired therapeutic resistance (11). Comprehensive pan-cancer analyses have revealed that CRC progression follows dual evolutionary trajectories involving chromosomal instability (CIN) and MSI (12). The coexistence of multiple subclones within a tumor can facilitate “covert” immune evasion mechanisms, including human leukocyte antigen (HLA) loss and β2-microglobulin (B2M) mutations. Notably, nearly 50% of MSS CRCs harbor such genomic-level immune defects (13). Furthermore, single-cell and spatial transcriptomic analyses have uncovered marked spatiotemporal heterogeneity between primary tumors and liver metastases, particularly in cancer stem cell proportions, cancer-associated fibroblast (CAF) states, and CD8^+^ T cell exhaustion (14, 15). Tumors from patients of different age groups also exhibit distinct immune microenvironments, skewed toward either immunosuppressive or chronic inflammatory profiles (16), underscoring the intricate relationship between ITH and immune ecology.

With advancements in computational methodologies, ITH can now be quantitatively assessed at both the DNA and RNA levels with increasing precision (17–19). The recently developed Dissecting Expression Profile-derived Tumor Heterogeneity 2 (DEPTH2) algorithm enables the estimation of ITH by evaluating variability in RNA-seq expression profiles (20). Validated across 33 cancer types, DEPTH2 has demonstrated strong correlations with genomic instability, poor clinical outcomes, and immunosuppressive tumor microenvironments (17). However, investigations of ITH in CRC remain largely limited to pan-cancer analyses or single-cohort studies, often lacking consideration of CRC-specific molecular subtypes and immune contexture. Furthermore, the absence of standardized scoring thresholds and external validation in independent cohorts hinders the clinical applicability of ITH-based metrics in CRC.

To address this critical gap, we integrated multicenter transcriptomic datasets from TCGA-COAD/READ and two independent GEO cohorts (GSE40967 and GSE87211) and adapted the DEPTH2 algorithm to develop a CRC-specific ITH score, establishing a standardized threshold for heterogeneity-based patient stratification. Based on 52 ITH-related genes (ITRGs) that were significantly associated with overall survival (OS), we evaluated 101 combinations of feature selection and modeling strategies and ultimately derived a robust nine-gene prognostic model, which was subsequently validated in both external cohorts. We systematically compared immune infiltration profiles, ICI response potential, and survival outcomes between high- and low-risk subgroups. Through SHapley Additive exPlanations (SHAP) analysis and both *in vitro* and *in vivo* functional assays, IL20RB was identified as a key molecular driver. The proposed integrated “ITH–immunity–prognosis” stratification framework offers a novel molecular rationale for precision subtyping and immunotherapeutic targeting in CRC, while establishing a mechanistic foundation for future IL20RB-focused clinical investigations.

## 2 Methods

### 2.1 Data acquisition

The training dataset was derived from The Cancer Genome Atlas (TCGA), encompassing two distinct cohorts: colon adenocarcinoma (COAD) and rectal adenocarcinoma (READ). RNA sequencing expression profiles, formatted as fragments per kilobase of transcript per million mapped reads (FPKM), together with corresponding somatic mutation data and clinical follow-up information, were downloaded from the Genomic Data Commons (GDC) Data Portal (https://portal.gdc.cancer.gov/). For independent validation, transcriptomic and clinical data were retrieved from two datasets, GSE40967 and GSE87211, available in the Gene Expression Omnibus (GEO, https://www.ncbi.nlm.nih.gov/geo/) database. For both GEO cohorts, raw microarray data were processed using robust multi-array average (RMA) and quantile normalization, and probe sets were mapped to official gene symbols. To mitigate platform-related differences between RNA-seq–based (TCGA) and microarray-based (GEO) expression data, gene expression values used for model construction and validation were log2-transformed and z score–standardized within each cohort.

### 2.2 Calculation of ITH scores and prognostic evaluation

ITH in CRC samples was quantified from RNA-seq expression profiles using the DEPTH2 algorithm, which calculates ITH scores by evaluating the dispersion of gene expression features at the single-sample level. OS time (futime) and survival status (fustat) were utilized to determine the optimal cut-off value for stratifying patients into high- and low-ITH groups. The optimal threshold for distinguishing high- and low-ITH subgroups was determined using the surv_cutpoint function implemented in the *survminer* R package. Kaplan–Meier (K-M) analysis combined with the log-rank test was employed to compare survival outcomes between the defined high- and low-ITH patient groups. Associations between ITH scores and clinicopathological variables were analyzed using the non-parametric Wilcoxon rank-sum test. Boxplots were created using the *ggpubr* package to visually represent the findings.

### 2.3 Differential expression analysis

To identify transcriptional differences, gene expression profiles were compared between patients in the high- and low-ITH categories. Low-abundance genes were first filtered out, after which gene expression levels were compared using the non-parametric Wilcoxon rank-sum test. Log_2_ fold change (log_2_FC) values were calculated, and differentially expressed ITRGs were identified based on the criteria |log_2_FC| > 1 and a false discovery rate (FDR) < 0.05, adjusted using the Benjamini–Hochberg method. The top 50 significantly upregulated and top 50 significantly downregulated differentially expressed genes (DEGs), ranked by FDR, were selected for visualization. A heatmap was generated to illustrate the expression patterns of these ITRGs between the high- and low-ITH subgroups.

### 2.4 Construction of multi-algorithm prognostic models based on ITRGs

Univariate Cox regression analysis was performed on ITRGs in the TCGA cohort to identify candidates significantly associated with OS, applying a significance threshold of *p* < 0.01. To refine prognostic markers and reduce data dimensionality, a suite of machine learning techniques was applied, including Least Absolute Shrinkage and Selection Operator (LASSO), Ridge regression, Elastic Net, Random Survival Forest (RSF), stepwise Cox regression, supervised principal components analysis (SuperPC), and partial least squares Cox regression (PLS-Cox). Prognostic models retaining fewer than five features were excluded from further evaluation. All models were trained on the TCGA dataset and externally validated in the GSE40967 and GSE87211 cohorts. Model performance was assessed using Harrell’ s concordance index (C-index), and models were ranked based on the average C-index across the TCGA and the two GEO datasets. The predictive performance and robustness of each model were visualized using the *ComplexHeatmap* package.

### 2.5 Model interpretability analysis

The best-performing prognostic model, identified based on the highest C-index, was selected for interpretability analysis. SHAP values were calculated for each feature using the *kernelshap* package. Feature importance was visualized with the *shapviz* package to evaluate the relative contribution of each variable. Various visualization methods—such as bar charts, beeswarm diagrams, waterfall graphs, and force plots—were used to display SHAP values and highlight interpretability. Features were ranked according to their average SHAP values, allowing for the identification of the most influential ITRGs driving model predictions.

### 2.6 Evaluation of predictive performance and independent prognostic value of the model

Using the prognostic model’s median risk score (RS) as a threshold, patients in the training and each validation dataset were categorized into high- and low-risk groups. Survival outcomes between the high- and low-risk groups were compared using K-M analysis, with statistical significance evaluated via the log-rank test. In the TCGA dataset, both the RS and clinical factors—such as age, sex, and TNM stage—were subjected to univariate and multivariate Cox regression analyses to assess their prognostic significance. Hazard ratios (HRs) and 95% confidence intervals (CIs) were calculated to determine the independent prognostic value of the RS, and a forest plot was constructed to visualize the contribution of each variable to survival outcomes. The model’ s predictive accuracy was further assessed via time-dependent receiver operating characteristic (ROC) curve analysis. To evaluate predictive performance, area under the curve (AUC) values were computed for 1-, 3-, and 5-year survival estimates in the training and validation cohorts and contrasted with those derived from standard clinical indicators.

### 2.7 Nomogram construction and validation

A nomogram was developed from the multivariate Cox model by combining the RS with key clinical features to predict 1-, 3-, and 5-year survival probabilities for patients in the TCGA cohort. The nomogram was generated using the *regplot* package. Personalized risk estimates were derived from the nomogram, and calibration plots were used to evaluate how well the predicted survival aligned with actual outcomes. Internal validation was conducted using 1000 bootstrap replicates. C-index was employed to assess the nomogram’s ability to distinguish between different survival outcomes.

### 2.8 Evaluation of immune microenvironment characteristics and functional status

To explore the association between the model-derived RS and the tumor immune microenvironment (TME), the ESTIMATE algorithm was applied to infer immune infiltration, stromal content, and overall tumor purity by calculating the Immune Score, Stromal Score, and composite ESTIMATE Score. These metrics were compared between high- and low-risk subgroups. Single-sample gene set enrichment analysis (ssGSEA) was conducted to assess immune functional states. Additionally, multiple immune cell infiltration estimation methods, including TIMER and CIBERSORT, were integrated to provide a comprehensive characterization of the immune landscape. Spearman correlation analysis was used to evaluate associations between the RS and the abundance of various immune cell subsets. Immune cell populations with statistically significant correlations (*p* < 0.05) were identified and visualized using boxplots and bubble plots.

### 2.9 Evaluation of immunotherapy response potential, immune checkpoint characteristics and genomic features

Expression profiles of immune checkpoint genes (ICGs) were extracted and compared between high- and low-risk groups using the Wilcoxon rank-sum test; statistical significance was defined as a FDR < 0.05. Differentially expressed ICGs were visualized using boxplots. To evaluate tumor immune evasion and the potential responsiveness to ICIs, Tumor Immune Dysfunction and Exclusion (TIDE) scores were obtained from the TIDE web portal (https://tide.dfci.harvard.edu), and their distributions between risk groups were compared and displayed as violin plots, providing an estimate of the model’ s predictive capacity for immunotherapy benefit. In the TCGA cohort, tumor mutational burden (TMB) was calculated from somatic mutation data as the number of nonsynonymous mutations per megabase. TMB levels were compared between high- and low-risk groups using the Wilcoxon rank-sum test, and the association between TMB and the RS was evaluated by Spearman correlation analysis. MSI status (MSS, MSI-L and MSI-H) was obtained from TCGA clinical annotations, and differences in RS among MSI subgroups were assessed. RNA-based stemness indices (RNAss) were retrieved from published TCGA pan-cancer stemness datasets (21) and correlated with the RS using Spearman analysis. Somatic mutation landscapes of canonical colorectal cancer driver genes (e.g. APC, TP53 and KRAS) in the high- and low-risk groups were summarized and visualized as waterfall plots using the *maftools* package.

### 2.10 Gene set enrichment analysis

To elucidate functional differences between the high- and low-risk groups, GSEA was performed using the *clusterProfiler* package, with reference to gene sets from the Kyoto Encyclopedia of Genes and Genomes (KEGG) and Gene Ontology (GO) collections within the Molecular Signatures Database (MSigDB). Only gene sets containing between 15 and 500 genes were included in the analysis. Statistical significance was defined as *p* < 0.05. Based on normalized enrichment scores (NES), significantly enriched pathways were independently identified for each risk group. Enrichment plots were generated to visualize the key biological processes and pathways associated with each subgroup.

### 2.11 Drug sensitivity prediction analysis

The *oncoPredict* package was applied to estimate drug sensitivity in TCGA samples, aiming to explore associations between RS generated by the model and potential therapeutic responses. The predictive model was trained using drug sensitivity profiles, represented by half-maximal inhibitory concentration (IC_50_) values, from the Genomics of Drug Sensitivity in Cancer 2 (GDSC2) database. Batch correction was applied to the TCGA test set to reduce technical variability, and genes within the lowest 20% of standard deviation were excluded to enhance prediction robustness. Predicted IC_50_ values were integrated with risk group classifications, and differences in drug sensitivity between high- and low-risk groups were assessed using the Wilcoxon rank-sum test. Statistical significance was defined as *p* < 0.001. Boxplots were generated to visualize variations in drug response across the two subgroups.

### 2.12 Cell culture and treatment

CRC cell lines (HCT116, HT29, SW480, and SW620) and the normal human colonic epithelial cell line (NCM460) were sourced from the Cell Bank of the Chinese Academy of Sciences. HCT116 and NCM460 cells were cultured in RPMI-1640 medium, while HT29, SW480, and SW620 cells were maintained in DMEM. All culture media were supplemented with 10% fetal bovine serum (FBS) and 1% penicillin–streptomycin (Gibco). Cells were incubated at 37°C in a humidified atmosphere containing 5% CO_2_ and subcultured regularly. To achieve knockdown of IL20RB expression, lentiviral vectors encoding IL20RB-specific short hairpin RNA (sh-IL20RB) and a negative control shRNA (sh-NC) were synthesized and packaged by Heyuan Biotechnology Co., Ltd. (Shanghai, China). HCT116 and SW480 cells were infected with the respective lentiviruses following the manufacturer’s instructions. Stable transfectants were selected using puromycin to generate cell lines with sustained gene silencing. The effectiveness of gene silencing was validated using both quantitative reverse transcription PCR (qRT-PCR) and Western blotting (WB).

### 2.13 qRT-PCR analysis

Total RNA was isolated using TRIzol reagent (Invitrogen), and its purity and concentration were evaluated with a NanoDrop 2000 spectrophotometer (Thermo Fisher Scientific). Only samples meeting quality criteria for purity and concentration were used for subsequent analyses. Complementary DNA (cDNA) was synthesized using the PrimeScript RT Reagent Kit (Takara). qRT-PCR was performed using SYBR Premix Ex Taq II (Takara) on an ABI 7500 Real-Time PCR System. Thermal cycling included an initial denaturation at 95°C for 30 seconds, followed by 40 amplification cycles of 95°C for 5 seconds and 60°C for 30 seconds. GAPDH served as the housekeeping gene, and relative expression was quantified using the 2^-ΔΔCt^ method. Primer sequences used in this analysis are listed in **Supplementary Table S1**.

### 2.14 WB analysis

Total protein was extracted from cell and tissue samples using RIPA lysis buffer (Beyotime, Shanghai), supplemented with 1% protease and phosphatase inhibitor cocktail. Lysates were centrifuged at 12,000 × *g* for 15 minutes at 4°C, and the supernatant was collected for protein quantification using the BCA Protein Assay Kit (Beyotime). Proteins were resolved by SDS-PAGE and subsequently transferred onto polyvinylidene difluoride (PVDF) membranes (Millipore) for immunoblotting. To block nonspecific binding, membranes were incubated with 5% non-fat milk at room temperature for 1 hour, followed by overnight incubation at 4°C with the appropriate primary antibodies. After washing with TBST, membranes were incubated for 1 hour at room temperature with HRP-conjugated goat anti-rabbit secondary antibodies. Protein bands were visualized using enhanced chemiluminescence (ECL) reagents (Thermo Fisher Scientific), and images were captured with the Tanon 5200 imaging system. Band intensity was quantified by densitometric analysis using ImageJ software. *β*-Actin was used as the internal loading control. The sources and catalog numbers of all primary and secondary antibodies are listed in **Supplementary Table S2**.

### 2.15 Immunohistochemistry and hematoxylin–eosin staining analysis

Human CRC tissues and paired adjacent normal tissues, along with murine tumor specimens, were fixed in 10% neutral-buffered formalin for 24–48 hours, followed by routine paraffin embedding and sectioning at a thickness of 4 μm. Tissue slides were incubated at 60°C for 2 hours, then cleared of paraffin using xylene and sequentially rehydrated through decreasing concentrations of ethanol. To block endogenous peroxidase activity, tissue sections were treated with 3% hydrogen peroxide solution. Antigen retrieval was carried out by heating the sections in sodium citrate buffer (pH 6.0) using a microwave, followed by a cooling period at room temperature. Sections were blocked with 5% normal bovine serum for 30 minutes and then incubated overnight at 4°C with primary antibodies targeting IL20RB (1:200, Proteintech) or Ki-67 (1:400, Abcam). Following PBS washes, the sections were incubated at room temperature for 1 hour with an HRP-linked goat anti-rabbit secondary antibody (1:5000, ZSGB-Bio). Chromogenic detection was conducted using 3,3′-diaminobenzidine (DAB; ZSGB-Bio), followed by hematoxylin counterstaining, dehydration, clearing, and mounting with neutral resin. Microscopic images were acquired using an Olympus BX53 imaging system, and five random high-power fields (400× magnification) per section were selected for analysis. Positive staining was semi-quantified as mean optical density (MOD) using ImageJ software. In parallel, selected tissue sections were subjected to HE staining following standard protocols to assess tissue architecture and morphological alterations under light microscopy.

### 2.16 Cell counting kit-8 cell proliferation assay

Cells were harvested by trypsinization, resuspended in complete culture medium, and counted. Cell suspensions were diluted to a final concentration of 5 × 10^4^ cells/mL, and 100 μL was dispensed into each well of a 96-well plate, with five replicates allocated per group. Plates were incubated overnight at 37°C in a humidified incubator containing 5% CO_2_ to allow for cell adherence. At 24, 48, and 72 hours post-seeding, 10% (v/v) CCK-8 reagent (Dojindo, Japan) was added to each well and incubated in the dark for 1 hour. The absorbance at 450 nm was recorded using a BioTek microplate reader (USA). Optical density (OD) values were averaged for each group and used to construct cell proliferation curves.

### 2.17 Transwell migration and invasion assays

Cells were harvested by trypsinization, resuspended in serum-free medium, counted, and adjusted to a final concentration of 5 × 10^4^ cells per 100 μL. For the migration assay, cell suspensions were seeded into the upper chambers of uncoated Transwell inserts (8 μm pore size, Corning). For the invasion assay, the membranes were pre-coated with a uniform layer of Matrigel (Corning) and incubated at 37°C for 2 hours to allow gel formation prior to cell seeding.

To create a chemoattractant gradient, 600 μL of complete medium supplemented with 20% FBS was added to the lower chambers.Following a 48-hour incubation at 37°C with 5% CO_2_, cells that had not migrated or invaded through the membrane were carefully removed from the upper surface using a cotton swab. The inserts were rinsed twice with PBS, fixed in 4% paraformaldehyde for 20 minutes, and subsequently stained with 0.1% crystal violet for 15 minutes. After rinsing and air-drying, five random high-power fields (400× magnification) per insert were imaged under a light microscope. The number of stained cells was quantified to assess migration and invasion capacity.

### 2.18 Wound healing assay

Cells were collected via trypsinization, resuspended in complete growth medium, and diluted to a final concentration of 5 × 10^5^ cells/mL. An equal amount of the prepared cell suspension was added to each well of a 6-well culture plate. After incubation at 37°C in a humidified 5% CO_2_ atmosphere until a confluent monolayer was established, Non-adherent and suspended cells were carefully removed by rinsing the wells twice with sterile PBS. A straight vertical scratch was created across each well using a sterile 200 μL pipette tip, maintaining consistent wound width across all wells. Following a second PBS wash to eliminate detached cells and debris, images of the wounded area were captured at 0 h. Cells were then cultured under standard conditions, and additional images were taken at designated time points (e.g., 24 h), preferably using the same field of view. Wound width was measured using ImageJ software, calibrated against an embedded 200 μm scale bar. The reduction in wound width between 0 h and the final time point was used to calculate the migration distance, serving as an indicator of cellular migratory capacity.

### 2.19 Subcutaneous tumor xenograft and *in vivo* imaging assay

Female BALB/c nude mice (aged 4–6 weeks) were obtained from Beijing Vital River Laboratory Animal Technology Co., Ltd. SW480 cells stably transfected with either sh-IL20RB or sh-NC were harvested by trypsinization, resuspended in serum-free medium, and adjusted to a final concentration of 5 × 10^6^ cells/mL. A total of 100 μL of the cell suspension was subcutaneously injected into the right axillary region of each mouse. Each experimental group (sh-IL20RB and sh-NC) consisted of three mice (*n* = 3 per group).

To evaluate *in vivo* tumor growth, bioluminescence imaging was performed on day 25 post-inoculation. D-luciferin (150 mg/kg, Promega) was administered via intraperitoneal injection 10 minutes before imaging using the IVIS Spectrum imaging system (PerkinElmer). Images were analyzed with Living Image software, and total bioluminescence intensity (photons/sec) was quantified to assess tumor metabolic activity.

### 2.20 Statistical analysis

Statistical analyses were conducted using R software (version 4.2.2). A *p* less than 0.05 was regarded as indicative of statistical significance, unless stated otherwise.

## 3 Results

### 3.1 Prognostic value and molecular expression features of the ITH score

**Figure 1:**
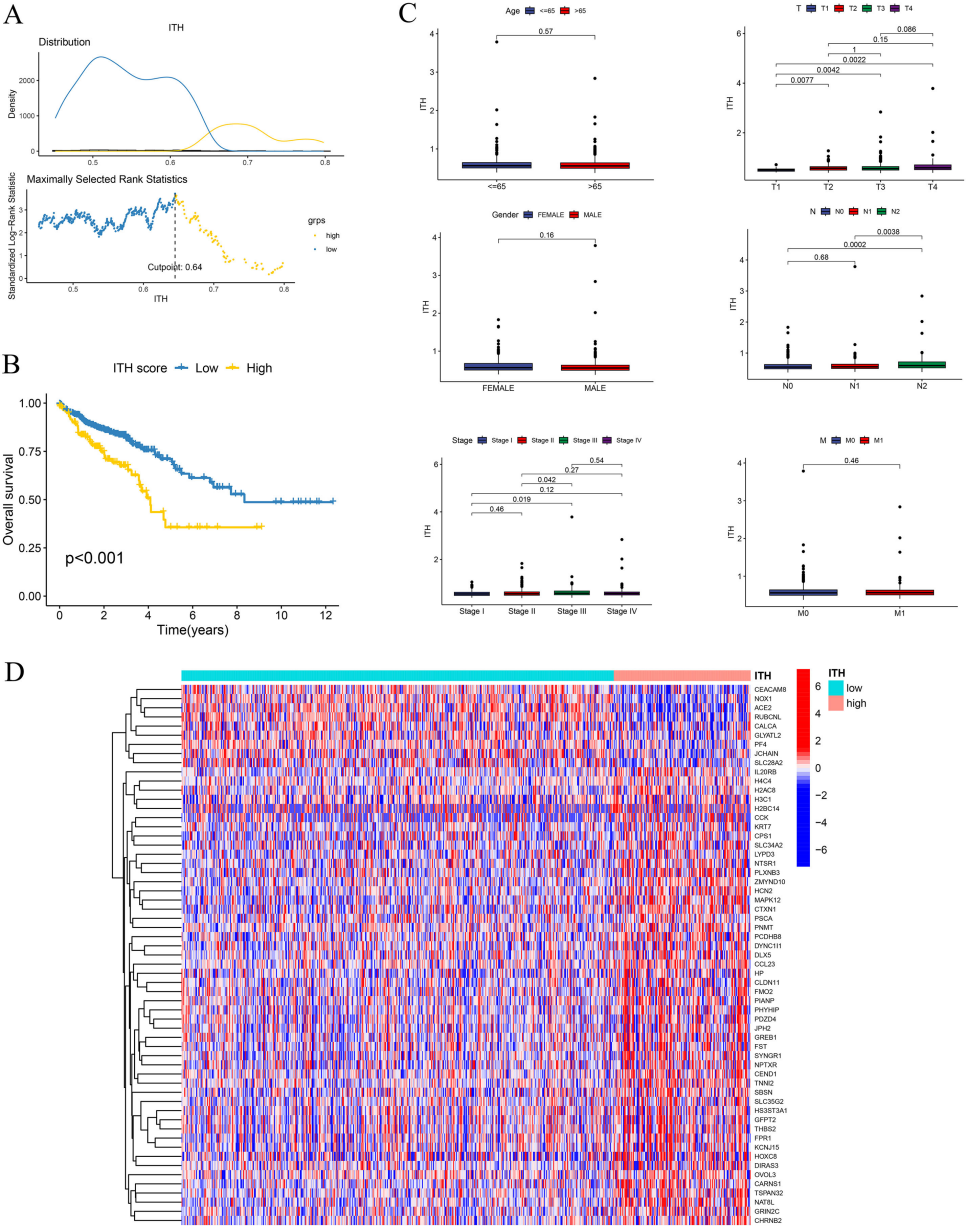
ITH score stratifies OS and correlates with clinical characteristics in CRC. **(A)** Upper panel: Density plot illustrating the distribution of ITH scores among CRC patients. Lower panel: Optimal cutoff value for ITH-based stratification determined using the maximally selected rank statistics method, dividing patients into low- and high-ITH groups. **(B)** K–M survival analysis showing significantly reduced OS in the high-ITH group compared to the low-ITH group (log-rank test, *p* < 0.001). **(C)** Box plots depicting associations between ITH scores and clinical variables (T stage, N stage, and AJCC stage). **(D)** Heatmap displaying the expression profiles of the top 100 differentially expressed genes between high- and low-ITH groups.

The ITH scores, derived using the DEPTH2 algorithm, exhibited a distinct bimodal distribution in the TCGA cohort (**Figure 1A**). An optimal cutoff value of 0.64 was identified using the maximal log-rank test and employed to stratify patients into high-ITH and low-ITH groups. Survival analysis using K-M curves showed that individuals in the high-ITH group experienced significantly shorter OS than those in the low-ITH group (**Figure 1B**), underscoring the prognostic stratification capability of the ITH score. Further analysis demonstrated significant variation in ITH scores across clinical subgroups: patients with T3 tumors had higher scores than those with T1 tumors, N1/N2 stages were associated with higher scores than N0, and AJCC Stage III patients had higher scores than Stage I (**Figure 1C**). In contrast, ITH scores showed no significant association with age, sex, or M stage.

Transcriptomic differential expression analysis identified a total of 324 ITRGs, comprising 315 upregulated and 9 downregulated genes in the high-ITH group. To visualize transcriptional differences, a heatmap was constructed displaying the top 50 most significantly altered genes between high- and low-ITH groups (**Figure 1D**).

### 3.2 Multiple machine learning approaches reveal key prognostic features

**Figure 2:**
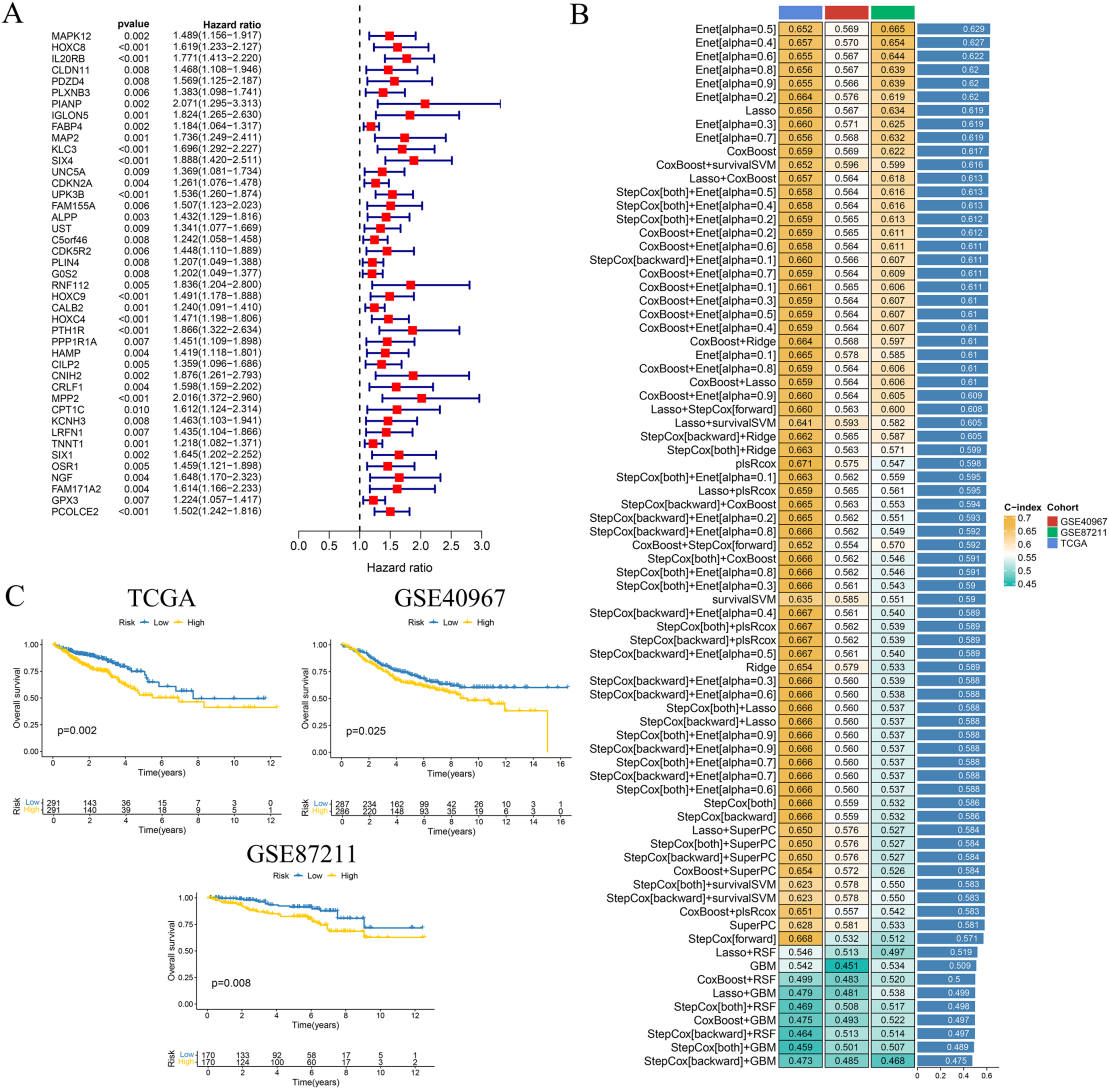
Construction and validation of an ITRG signature in CRC. **(A)** Forest plot of univariate Cox regression analysis identifying 52 ITRGs significantly associated with OS in the TCGA-CRC cohort. **(B)** Heatmap summarizing C-index for 101 combinations of feature selection and modeling algorithms based on ITRGs across the TCGA, GSE40967, and GSE87211 cohorts. The Elastic Net model with α = 0.5 achieved the highest average C-index and was selected as the final nine-gene prognostic signature. **(C)** K–M survival curves showing OS differences between high- and low-risk groups in the TCGA (left, p = 0.002), GSE40967 (middle, p = 0.025), and GSE87211 (right, p = 0.008) cohorts.

Univariate Cox regression analysis of the ITRGs revealed 52 genes significantly linked to OS at a threshold of *p* < 0.01. The majority of these genes exhibited HRs greater than 1, suggesting that elevated expression levels were correlated with poorer prognosis (**Figure 2A**).

To develop a robust prognostic model, we systematically evaluated 101 combinations of widely used machine learning algorithms, incorporating various feature selection and modeling strategies. Model performance was assessed using C-index across both the TCGA training cohort and the GEO validation cohort. The model constructed using Elastic Net feature selection (α = 0.5) demonstrated the highest predictive accuracy and strongest cross-cohort consistency, and was therefore selected as the optimal model (**Figure 2B**).

### 3.3 Interpretability analysis reveals key prognostic genes

**Figure 3:**
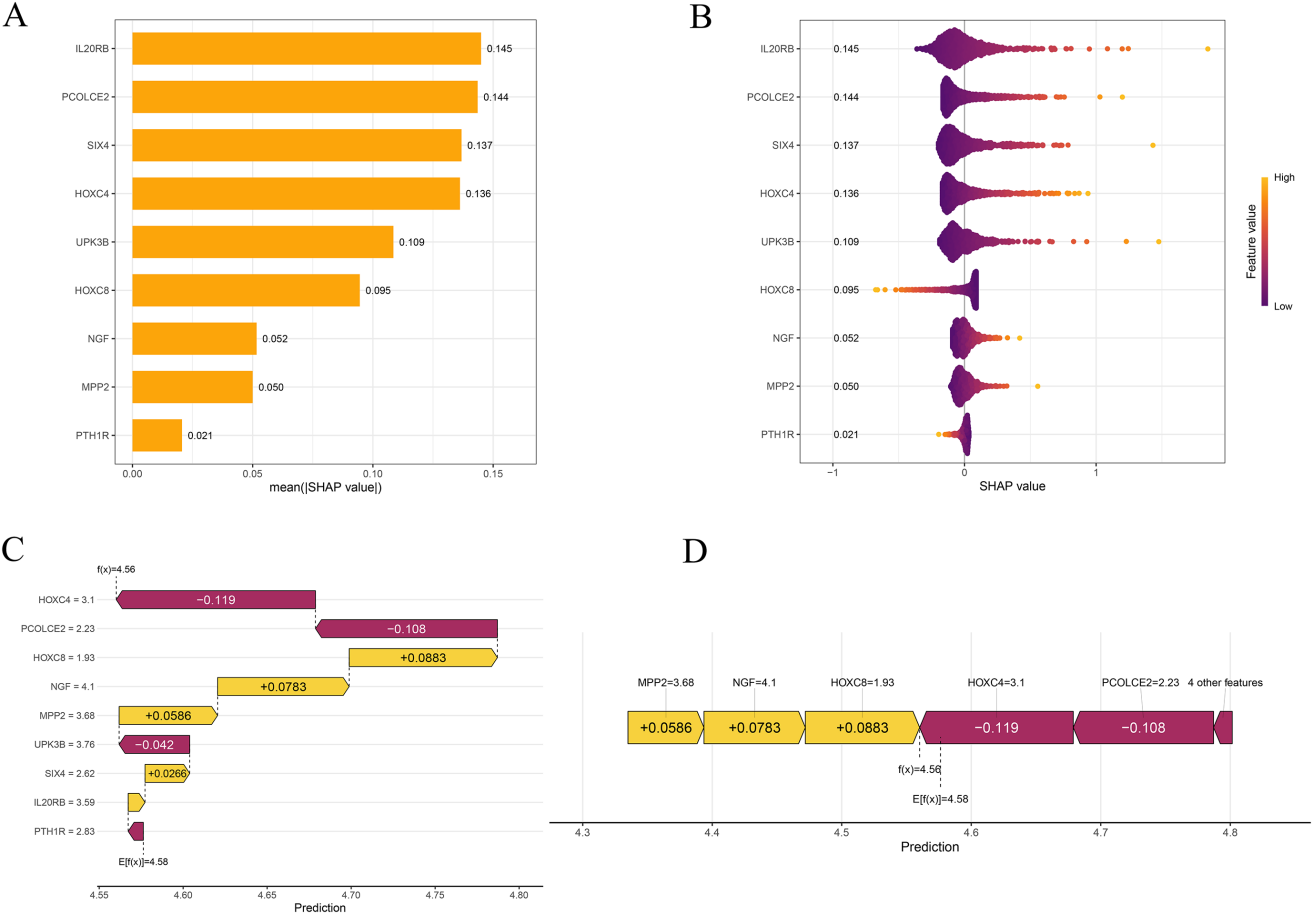
SHAP analysis reveals key contributors among ITRGs. **(A)** Bar plot displaying the mean absolute SHAP values of all 9 ITRGs, indicating their relative contributions to the model’s risk prediction. **(B)** SHAP summary plot showing the distribution of SHAP values for each ITRG. Feature values are color-coded by expression level (purple: low; yellow: high). **(C)** SHAP force plot illustrating the contribution of individual ITRGs to the predicted RS for a representative patient. **(D)** SHAP decision plot tracing the cumulative impact of all 12 ITRGs on the final prediction, from the model baseline expectation (E[f(x)]) to the individualized prediction (f(x)).

To elucidate how the prognostic model generates its risk predictions, we applied the SHAP framework to quantify the contribution of each gene to the model output. In the global feature importance ranking, IL20RB showed the largest mean absolute SHAP value (0.145), followed closely by PCOLCE2 (0.144), SIX4 (0.137), HOXC4 (0.136), UPK3B (0.109), HOXC8 (0.095), NGF (0.052), MPP2 (0.050) and PTH1R (0.021) (**Figure 3A**). The SHAP beeswarm plot indicated that high expression of IL20RB, PCOLCE2, SIX4, HOXC4 and UPK3B was predominantly associated with positive SHAP values, highlighting their roles as risk-promoting features in the model (**Figure 3B**). In addition, individual-level waterfall and force plots visualized how these genes jointly drove the predicted risk upward or downward in representative patients, thereby further improving the transparency and interpretability of the model’s predictions (**Figures 3C–D**).

### 3.4 Evaluation of predictive performance and independent prognostic value of the risk model

**Figure 4:**
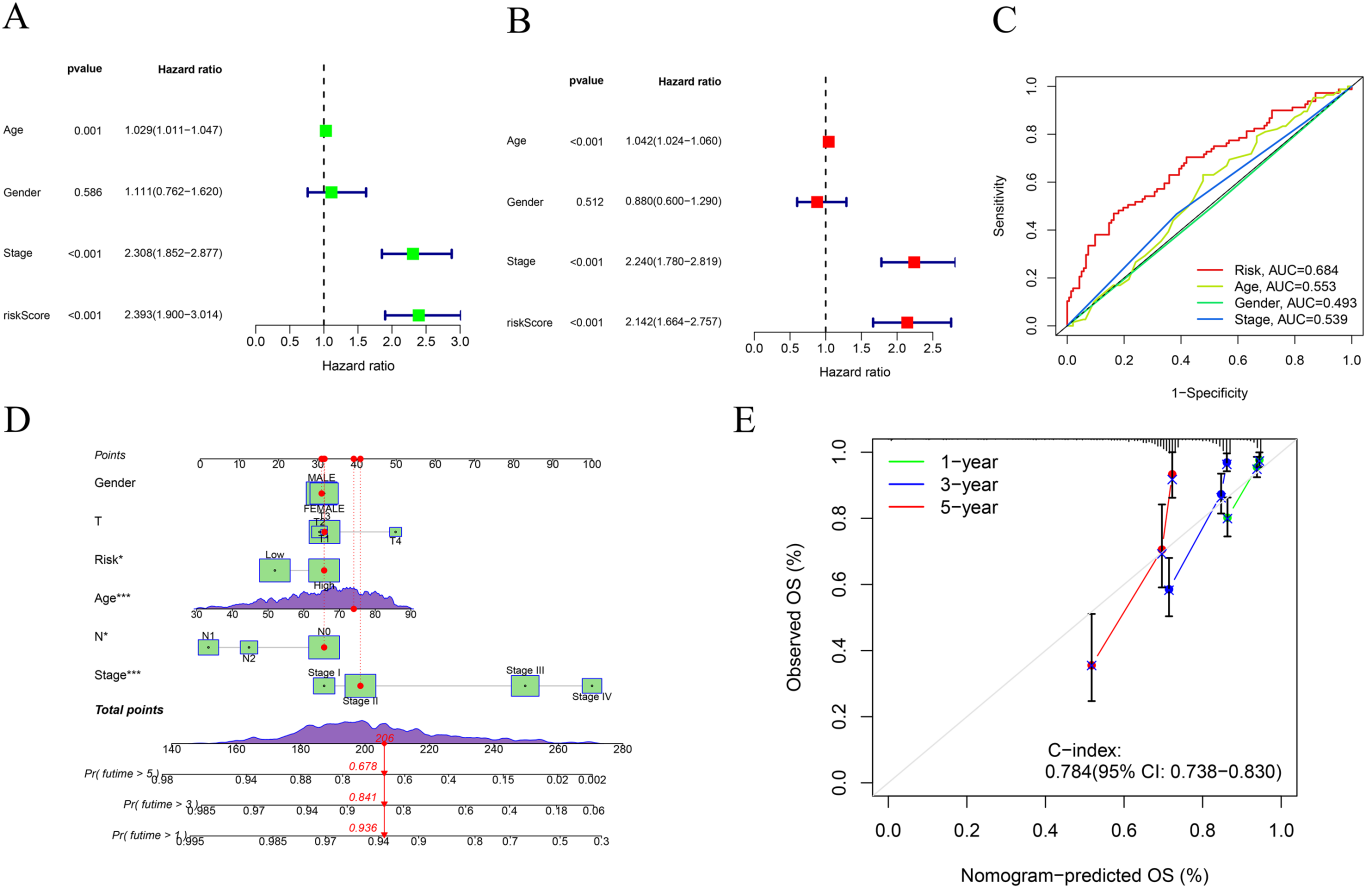
Independent prognostic value and clinical utility of the ITRG-based risk model in CRC. **(A, B)** Univariate **(A)** and multivariate **(B)** Cox regression analyses of OS in the TCGA cohort, incorporating age, gender, clinical stage, and RS. The ITRG-based RS remained an independent prognostic factor (*p* < 0.001). **(C)** Receiver operating characteristic (ROC) curves comparing the predictive accuracy (area under the curve, AUC) of the RS with conventional clinical variables (age, sex, and stage). The RS achieved the highest AUC (0.684). **(D)** Nomogram integrating the RS with clinical variables (sex, T stage, age, N stage, and overall stage) to estimate 1-, 3-, and 5-year OS probabilities in individual patients. **(E)** Calibration curves of the nomogram for 1-, 3-, and 5-year OS, showing good agreement between predicted and observed survival. The model demonstrated strong discriminative ability (C-index = 0.784; 95% CI, 0.738–0.830).

K–M survival curves in the TCGA training cohort and in both external validation cohorts (GSE40967 and GSE87211) consistently showed that patients classified as high-risk by the ITRG-based RS had significantly shorter OS than those in the low-risk group (**Figure 2C**), confirming the robustness of the model’s prognostic capability across datasets. Consistently, across all three cohorts, the nine signature genes were predominantly upregulated in the high-risk group, and patients ranked by increasing RS displayed a smooth transition from low- to high-risk status accompanied by an accumulation of death events (**Supplementary Figures S1A–C**). In the TCGA cohort, univariate and multivariate Cox regression analyses further demonstrated that the RS served as an independent prognostic indicator after adjustment for age, sex, and clinical stage (**Figures 4A–B**). Notably, the RS outperformed conventional clinical parameters in discriminative ability, as evidenced by its higher AUC compared with age, sex, and stage (**Figure 4C**). Time-dependent ROC analyses were then performed separately in each cohort to quantify the predictive accuracy of the model. In the TCGA training cohort, the prognostic signature yielded AUCs of 0.669, 0.664, and 0.645 for predicting 1-, 3-, and 5-year OS, respectively. In the GSE40967 validation cohort, the corresponding AUCs were 0.544, 0.565, and 0.573, whereas in the GSE87211 cohort they were 0.744, 0.709, and 0.648 (**Supplementary Figure S1D**).

### 3.5 Evaluation of the prognostic performance of the integrated clinical model

To enhance the accuracy of long-term prognostic assessment in CRC patients, we constructed a nomogram integrating the model-derived RS with key clinical parameters (**Figure 4D**). This tool enables visual quantification of each variable’s contribution to OS and facilitates individualized prediction of 1-, 3-, and 5-year survival probabilities. Calibration curves demonstrated excellent concordance between predicted and observed outcomes at all time points, reflecting strong predictive consistency (**Figure 4E**). The model achieved a C-index of 0.784 (95% CI: 0.738–0.830), further confirming its high discriminative power and robustness in survival risk stratification, underscoring its potential for clinical implementation.

### 3.6 Immune microenvironment characteristics and functional heterogeneity across risk groups

**Figure 5:**
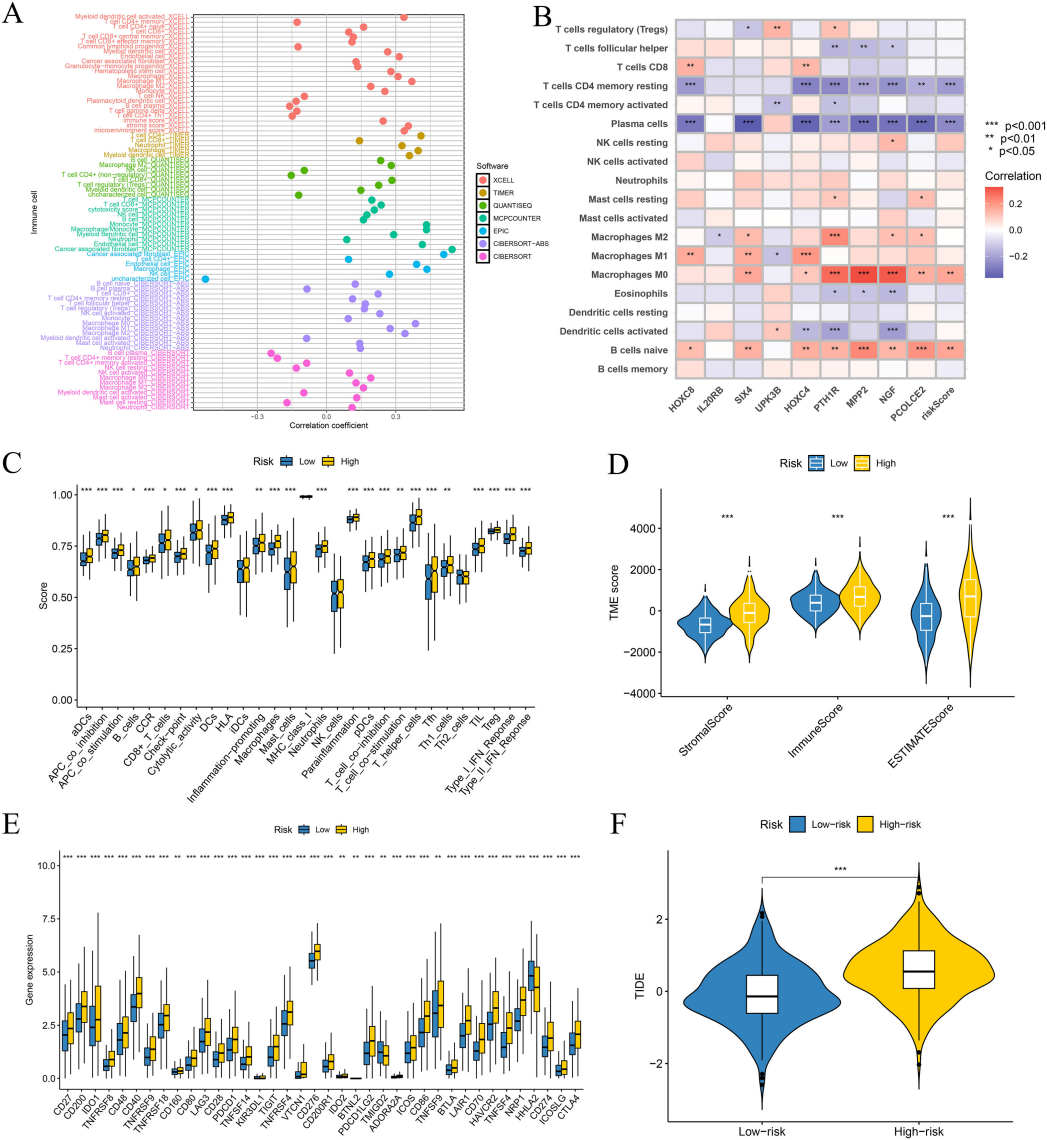
Immune landscape and immunotherapy relevance of the ITRG-based risk model. **(A)** Correlations between the ITRG-based RS and immune cell infiltration estimated by multiple deconvolution algorithms. Each point represents the correlation coefficient for a given immune cell type in a specific algorithm. **(B)** Heatmap showing Spearman correlations between the RS, individual signature genes and CIBERSORT-estimated immune cell fractions. **(C)** Boxplots of ssGSEA-derived immune-related functional pathway scores comparing high- and low-risk groups. **(D)** Violin plots comparing StromalScore, ImmuneScore and ESTIMATEScore between high- and low-risk groups, indicating a more immune- and stroma-rich microenvironment in the high-risk subgroup. **(E)** Boxplots showing differential expression of immune checkpoint–related genes between high- and low-risk groups. **(F)** Violin plots of TIDE scores in the two risk groups, with higher scores in the high-risk group indicating greater immune escape potential and a lower predicted benefit from immune checkpoint blockade. *p < 0.05, **p < 0.01, ***p < 0.001.

Immune infiltration analysis revealed that the high-risk group exhibited a characteristic “inflammatory-stromal” phenotype, marked by significant enrichment of CAFs, monocytes/macrophages and endothelial cells, together with a notable depletion of adaptive immune populations, including plasma cells and resting memory CD4^+^ T cells (**Figure 5A**). Correlation analysis between the signature genes, the ITRG-based RS and CIBERSORT-estimated immune cell fractions further supported this pattern: the RS was positively associated with macrophage subsets (M0, M1 and M2) and negatively associated with CD8^+^ T cells, resting memory CD4^+^ T cells, plasma cells and memory B cells (**Figure 5B**). Several genes within the signature, such as NGF, PCOLCE2 and PTH1R, displayed correlation profiles similar to those of the RS, whereas IL20RB itself showed only a weak association with M2 macrophages and no strong correlations with most other immune cell subsets, suggesting that the immunosuppressive milieu is mainly captured at the level of the integrated ITRG signature rather than by IL20RB alone. ssGSEA further indicated activation of a broad range of immune-related pathways in the high-risk group, including antigen processing and presentation, cytotoxic effector functions, Th1/Th2/Treg differentiation and interferon signaling, reflecting a dysregulated immune state with concomitant inflammatory activation and immunosuppressive activity (**Figure 5C**). Consistently, ESTIMATE analysis demonstrated significantly higher Immune Scores and Stromal Scores in the high-risk group, indicating substantial infiltration of immune and stromal components within the tumor microenvironment (**Figure 5D**).

### 3.7 Differences in immune checkpoint expression, immune escape and genomic features between risk groups

The high-risk group exhibited significant upregulation of a broad spectrum of immune checkpoint molecules, including both co-stimulatory and co-inhibitory targets. Notably elevated were CTLA4, CD274 (PD-L1), PDCD1LG2, LAG3, HAVCR2 (TIM-3), TIGIT, BTLA and CD276, as well as TNFR superfamily members such as OX40 (TNFRSF4), CD30 (TNFRSF8), 4-1BB (TNFRSF9) and GITR (TNFRSF18). In addition, metabolically associated immunosuppressive markers IDO1/2 and ADORA2A were markedly increased, consistent with a classical T-cell exhaustion phenotype (**Figure 5E**). The high-risk group also showed significantly higher TIDE scores than the low-risk group, indicating enhanced immune escape potential despite the apparently inflamed microenvironment (**Figure 5F**). In the TCGA cohort, high-risk tumors also displayed greater TMB, and the RS was positively correlated with TMB (**Supplementary Figure S2A**), whereas the RNAss was moderately negatively correlated with the RS (**Supplementary Figure S2C**). Moreover, MSI-L and MSI-H tumors tended to have higher RS than MSS tumors (**Supplementary Figure S2B**). Consistent with these observations, the somatic mutation landscapes of canonical colorectal driver genes such as APC, TP53 and KRAS were broadly similar between the high- and low-risk groups, without a consistent enrichment of specific driver alterations in either subgroup (**Supplementary Figure S2D–E**).

### 3.8 GSEA analysis reveals pathway and functional differences between risk subgroups

**Figure 6:**
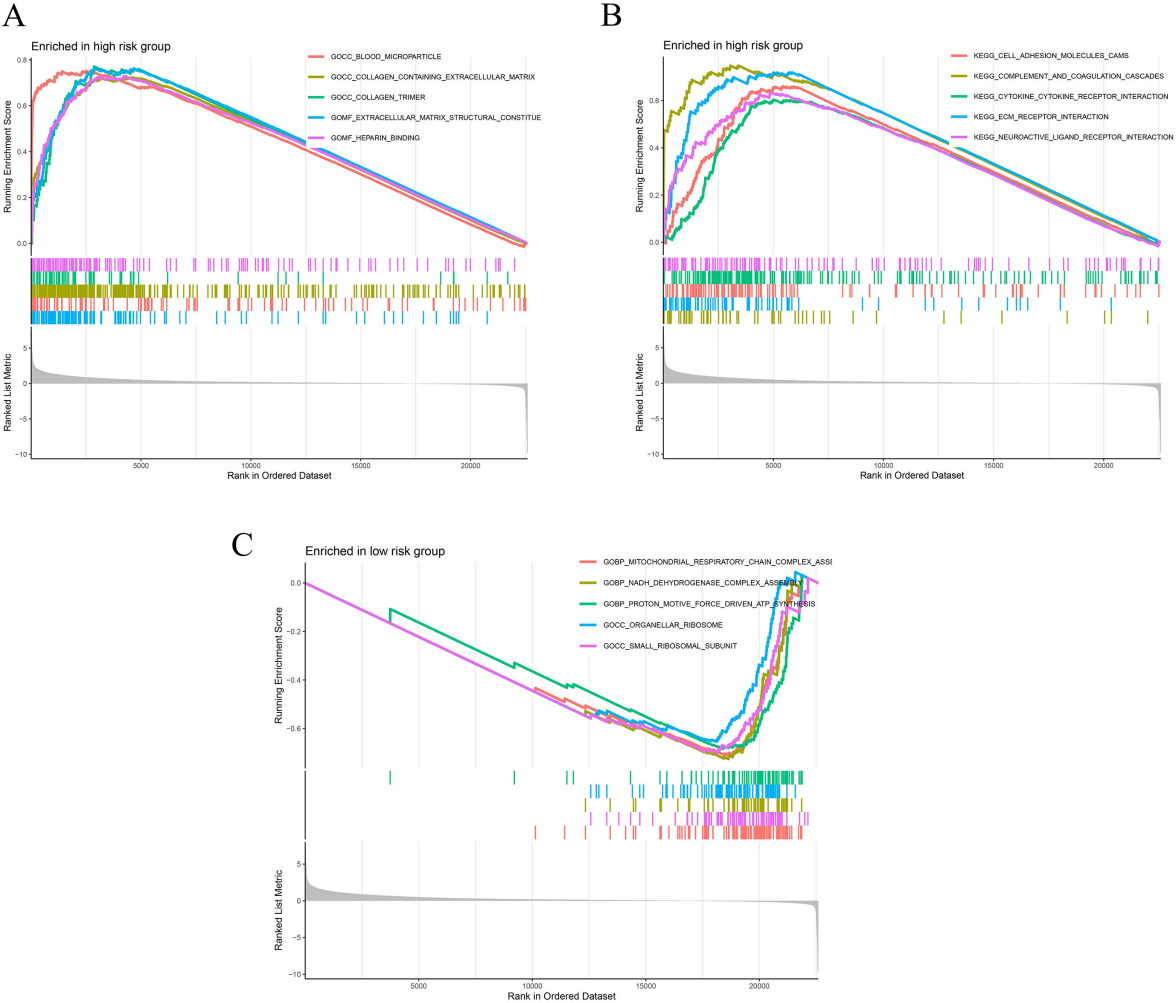
Functional enrichment analysis of ITRG-based risk groups using GSEA. **(A)** GO enrichment analysis in the high-risk group, highlighting pathways associated with extracellular matrix organization, immune regulation, and stromal activation. **(B)** KEGG pathway enrichment analysis in the high-risk group, showing significant enrichment in cytokine–cytokine receptor interaction, cell adhesion, and inflammatory signaling pathways. **(C)** GO enrichment analysis in the low-risk group, indicating upregulation of metabolic and biosynthetic processes, including oxidative phosphorylation and mitochondrial function.

GSEA indicated that high-risk patients exhibited significant enrichment in KEGG pathways related to immune activation and tissue remodeling. These included cell adhesion, extracellular matrix organization, complement and coagulation cascades, cytokine–cytokine receptor interaction, and neuroactive ligand–receptor interaction (**Figure 6B**). At the GO level, enrichment was observed in terms such as collagen-containing extracellular matrix, circulating blood microparticles, and heparin binding, collectively supporting the presence of an active inflammatory–stromal phenotype (**Figure 6A**). In contrast, the low-risk group showed predominant enrichment in pathways associated with energy metabolism and protein biosynthesis. These included mitochondrial respiratory chain complex assembly, ATP synthesis, and the functions of both ribosomal and mitochondrial ribosomal proteins (**Figure 6C**). These features suggest a metabolically active tumor microenvironment phenotype characterized by enhanced oxidative phosphorylation and translational activity.

### 3.9 Risk stratification and differences in drug sensitivity

**Figure 7:**
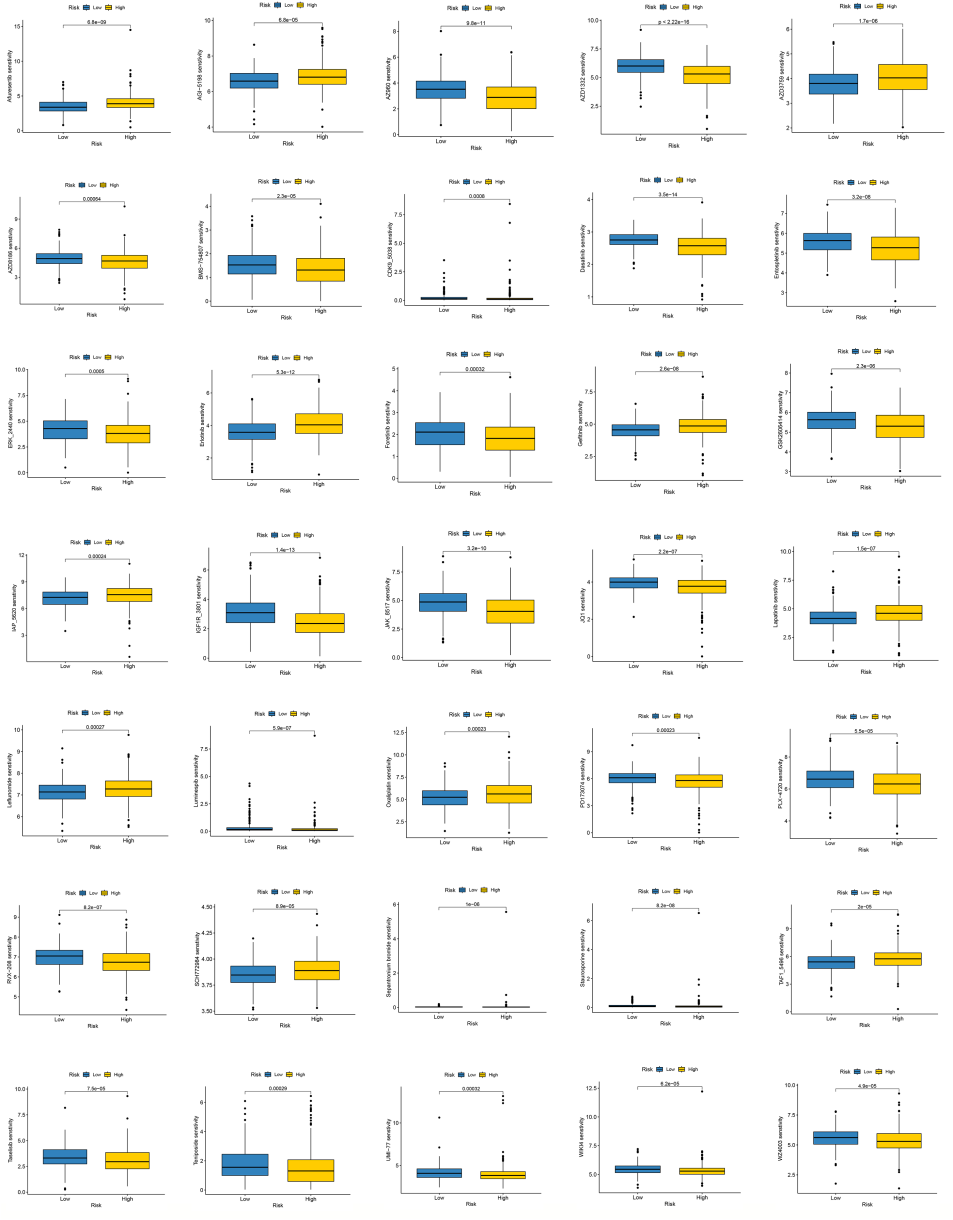
Drug sensitivity analysis between high- and low-risk groups based on the ITRG model. Box plots depict the predicted IC_50_ values for multiple therapeutic agents in the high- and low-risk subgroups.

Drug sensitivity prediction using the *oncoPredict* algorithm revealed that the low-risk group exhibited significantly lower estimated IC_50_ values for several therapeutic agents, including AKT/PI3K pathway inhibitors (Afuresertib, AZD8186), EGFR/HER2 inhibitors (Gefitinib, Erlotinib, Lapatinib, AZD3759), and the standard chemotherapeutic agent Oxaliplatin. These findings suggest a higher predicted sensitivity to these agents in the low-risk subgroup. In contrast, the high-risk group showed increased predicted sensitivity to multi-target kinase inhibitors (PD173074, Dasatinib, BMS-754807, PLX-4720) and the topoisomerase II inhibitor Teniposide, potentially reflecting a greater dependence on kinase signaling pathways and DNA damage response mechanisms (**Figure 7**).

### 3.10 High expression of IL20RB in CRC

**Figure 8:**
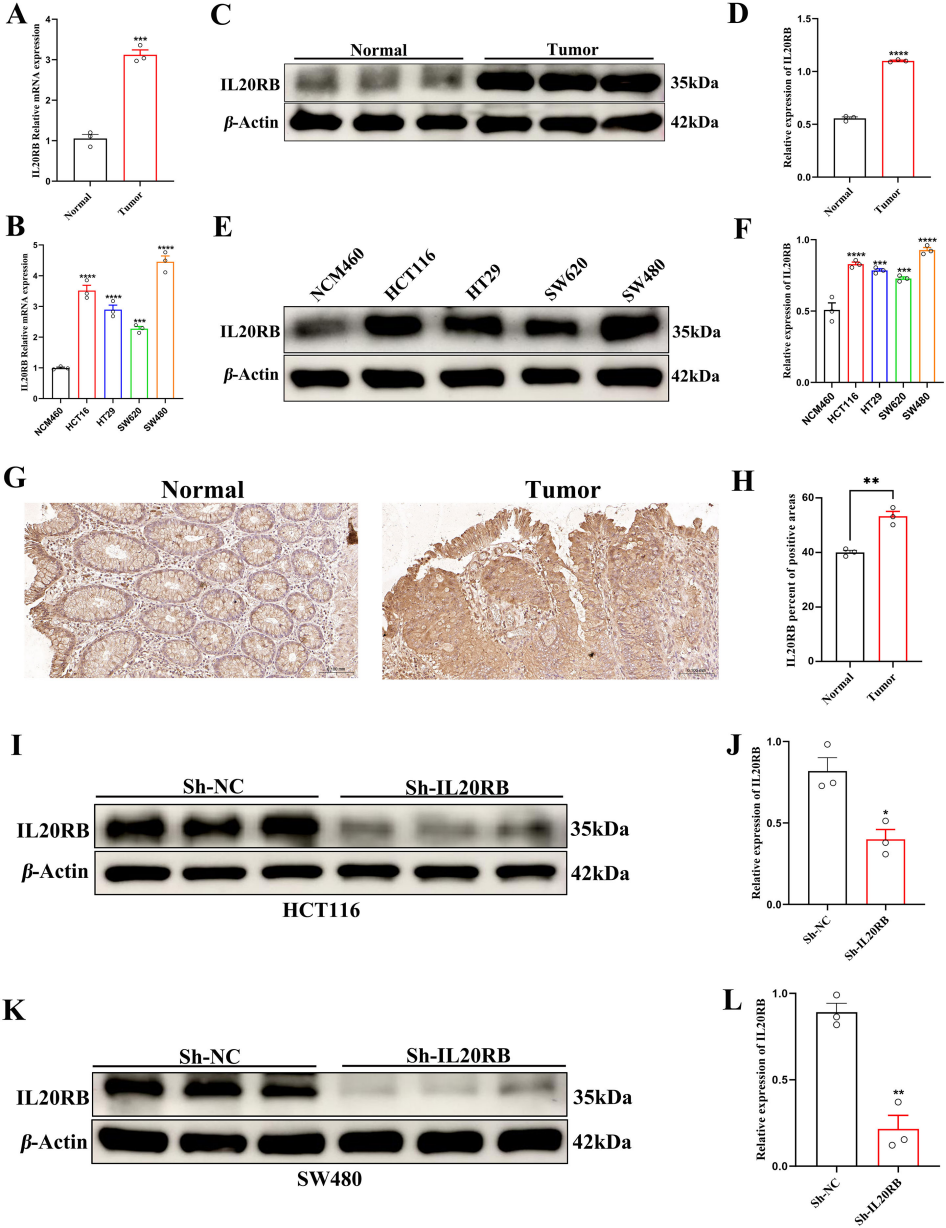
IL20RB is overexpressed in CRC tissues and cell lines and is efficiently silenced by shRNA. **(A)** Relative IL20RB mRNA expression in primary CRC tissues and matched adjacent normal mucosa as determined by qRT-PCR. **(B)** Baseline IL20RB mRNA levels in the normal colonic epithelial cell line NCM460 and CRC cell lines (HCT116, HT29, SW620 and SW480). **(C, D)** Representative WB and densitometric quantification of IL20RB protein expression in paired normal and tumor tissues; β-Actin served as the loading control. **(E, F)** WB analysis and quantitative assessment of IL20RB protein abundance in NCM460 and CRC cell lines. **(G, H)** Representative IHC staining for IL20RB in normal colonic mucosa and CRC tissues and semi-quantitative evaluation of staining intensity (mean optical density). **(I, J)** WB and densitometric analysis confirming efficient IL20RB knockdown in HCT116 cells stably transduced with sh-IL20RB compared with sh-NC controls. **(K, L)** Western blot and densitometric analysis showing analogous suppression of IL20RB expression in SW480 cells following sh-IL20RB transduction. Data are presented as mean ± SD; *p < 0.05, **p < 0.01, ***p < 0.001, ****p < 0.0001.

qRT-PCR analysis showed that IL20RB mRNA levels were significantly higher in CRC tissues than in matched adjacent normal tissues (**Figure 8A**), and were markedly elevated in CRC cell lines compared with the normal colonic epithelial cell line NCM460 (**Figure 8B**). Consistently, WB confirmed increased IL20RB protein expression in tumor tissues (**Figures 8C, D**) and across CRC cell lines (**Figures 8E, F**). Immunohistochemistry and semi-quantitative analysis further demonstrated a higher proportion of IL20RB-positive staining in tumor epithelium than in normal mucosa (**Figures 8G, H**), supporting its potential involvement in colorectal tumorigenesis.

### 3.11 Downregulation of IL20RB suppresses the proliferation, migration, and invasion of CRC cells

**Figure 9:**
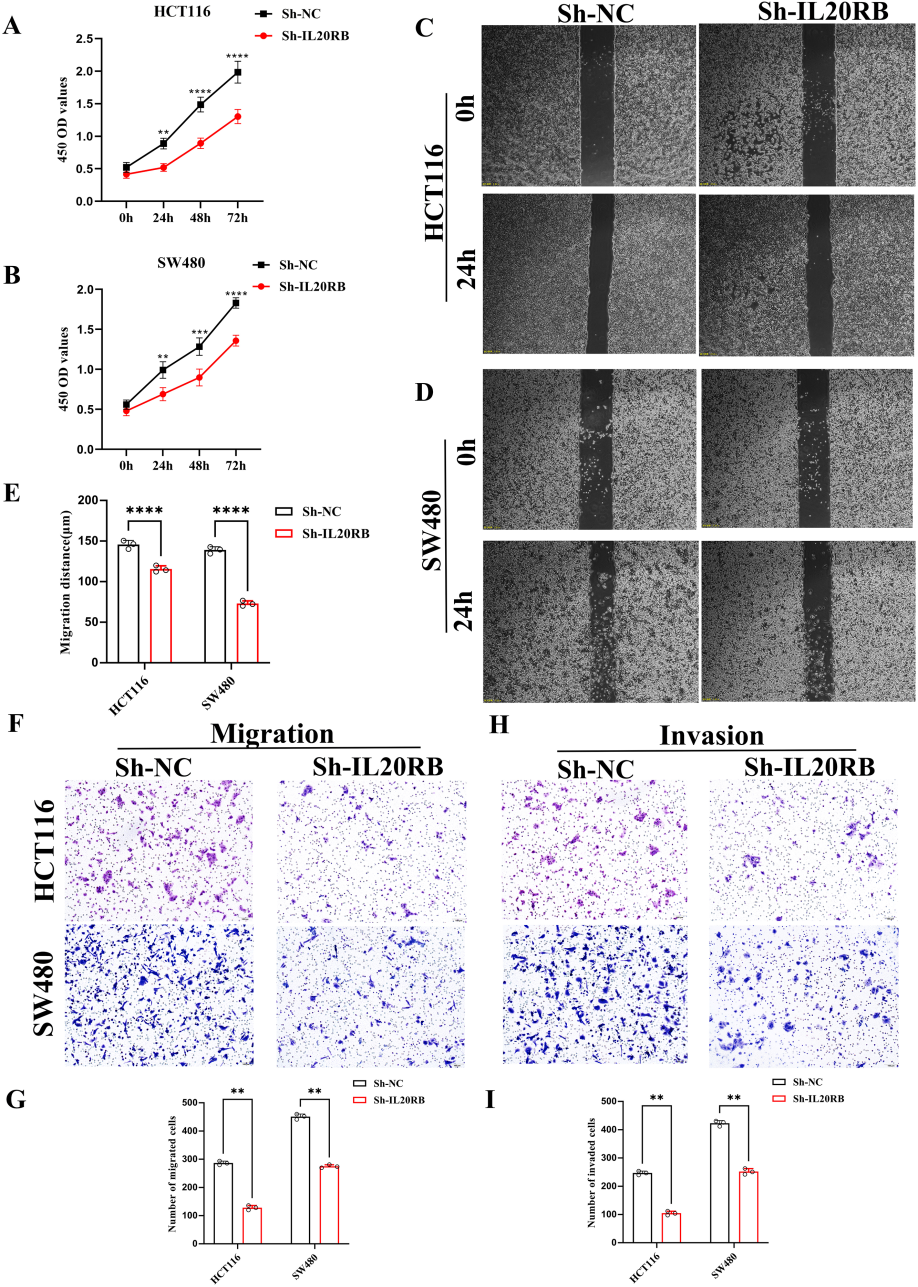
IL20RB knockdown inhibits proliferation, migration, and invasion of CRC cells. **<** CCK-8 assays showing that IL20RB knockdown significantly decreases the viability of HCT116 and SW480 cells at 24, 48, and 72 h, as reflected by reduced absorbance at 450 nm in the sh-IL20RB groups compared with sh-NC controls. **(C–E)** Representative wound-healing images and quantitative analysis demonstrating that IL20RB silencing markedly impairs migratory capacity in HCT116 and SW480 cells, as indicated by reduced wound closure over time in the sh-IL20RB groups. **(F–G)** Transwell migration assays illustrating a significant reduction in the number of migrated cells in both cell lines following IL20RB knockdown. **(H–I)** Transwell invasion assays showing that IL20RB depletion substantially diminishes the invasive potential of HCT116 and SW480 cells. Data are presented as mean ± SD; **p < 0.01, ***p < 0.001, ****p < 0.0001.

Stable IL20RB-knockdown cell lines were generated in HCT116 and SW480 cells via shRNA transfection. WB confirmed a marked reduction of IL20RB protein expression in both cell lines, indicating successful gene silencing (**Figures 8I–L**). CCK-8 assays showed that IL20RB knockdown significantly inhibited cell proliferation at 24, 48, and 72 hours (**Figures 9A–B**). Wound-healing assays demonstrated a pronounced decrease in migratory capacity in IL20RB-silenced cells (**Figures 9C–E**). Consistently, Transwell migration and invasion assays further revealed that IL20RB downregulation significantly reduced both migratory and invasive behavior, indicating a strong suppressive effect on the metastatic potential of CRC cells (**Figures 9F–I**).

### 3.12 Downregulation of IL20RB inhibits *in vivo* tumor growth of CRC cells

**Figure 10:**
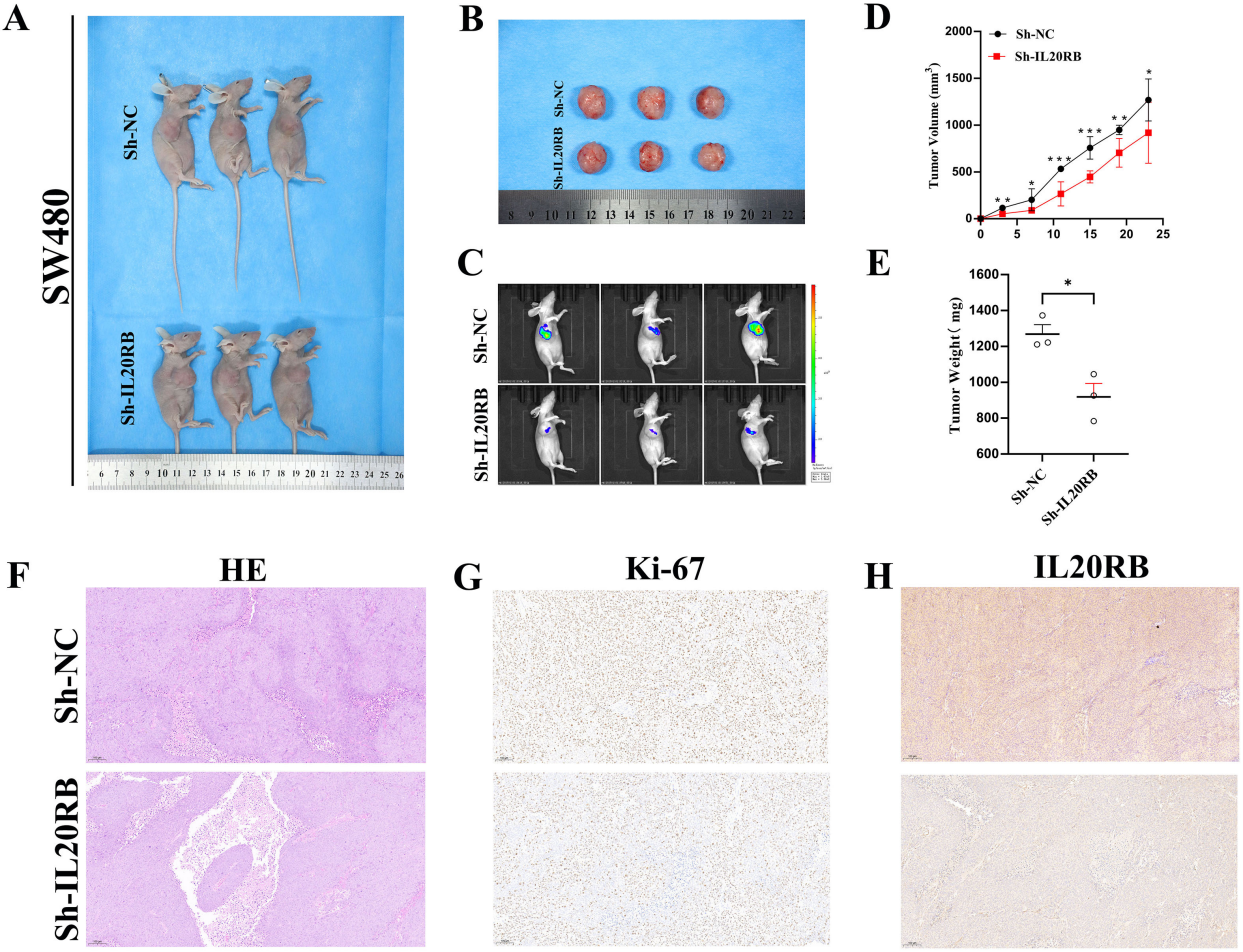
IL20RB knockdown suppresses tumor growth in vivo. **(A, B)** Representative photographs of nude mice and excised subcutaneous xenograft tumors generated from SW480 cells transduced with sh-NC or sh-IL20RB. **(C)** In vivo bioluminescence imaging showing reduced tumor burden in the sh-IL20RB group compared with the sh-NC group. **(D, E)** Tumor growth curves and endpoint tumor weights demonstrating significant inhibition of tumor progression following IL20RB knockdown (p < 0.05). **(F–H)** Representative histopathological and immunohistochemical images of xenograft tumors: H&E staining **(F)**, Ki-67 staining showing a decreased proliferative index **(G)**, and IL20RB staining confirming reduced IL20RB expression **(H)** in the sh-IL20RB group relative to controls. Data are presented as mean ± SD; *p < 0.05, **p < 0.01, ***p < 0.001.

In a subcutaneous xenograft model using SW480 cells in BALB/c nude mice, tumors in the sh-IL20RB group exhibited significantly reduced volumes and slower growth rates compared to those in the control group (**Figures 10A–B**). Analysis of tumor growth dynamics and endpoint tumor weights provided additional evidence that IL20RB knockdown significantly hindered tumor development (**Figures 10D–E**). *In vivo* bioluminescence imaging (IVIS) revealed markedly decreased signal intensity at tumor sites in the sh-IL20RB group, indicating reduced metabolic activity (**Figure 10C**). Hematoxylin and eosin (HE) staining showed no notable morphological abnormalities (**Figure 10F**), while Ki-67 immunostaining demonstrated a lower proliferation index in IL20RB-silenced tumors (**Figure 10G**). IHC analysis further confirmed reduced IL20RB protein expression *in vivo* (**Figure 10H**). Collectively, these results indicate that IL20RB downregulation significantly suppresses the tumorigenic potential of CRC cells *in vivo*.

## 4 Discussion

ITH refers to the genetic, transcriptional, and phenotypic variability among cancer cells within a single tumor (22). Extensive evidence has established ITH as a critical driver of tumor progression, immune evasion, and therapeutic resistance, largely through its influence on the evolving TME (23). Traditionally, ITH has been quantified using multi-region sequencing or single-cell omics technologies (24). While these methods provide high-resolution insights, their widespread clinical adoption remains limited due to high costs and technical complexity. The advent of the DEPTH algorithm, and its improved version DEPTH2, has introduced a novel transcriptome-based strategy for assessing ITH. By modeling gene expression dispersion from bulk RNA-seq data, these algorithms enable robust estimation of ITH without the need for matched normal tissues, thereby enhancing feasibility for broader clinical application (20). This is particularly relevant in CRC, a malignancy with pronounced heterogeneity, where conventional TNM staging and molecular classification systems often fail to fully account for the observed variability in clinical outcomes and treatment responses among patients with the same stage of disease (25, 26). Consequently, the integration of ITH as a novel prognostic biomarker may offer significant improvements in individualized risk stratification.

In the TCGA-CRC cohort, the ITH score calculated using the DEPTH2 algorithm exhibited a characteristic bimodal distribution. Applying a cutoff value of 0.64 enabled the stratification of patients into high- and low-ITH groups, revealing a significant difference in OS between these subgroups. A comprehensive pan-cancer analysis across 33 TCGA cancer types further confirmed that elevated ITH scores were consistently associated with more advanced clinical stages, increased genomic instability, and a more immunosuppressive tumor microenvironment (17, 20, 27). These findings are supported by external studies. For instance, in a cohort of 6,500 breast cancer patients, higher DEPTH2 scores were predominantly observed in triple-negative and HER2-positive subtypes and were significantly associated with proliferative signaling and diverse immune cell infiltrates (28). In CRC specifically, high ITH scores were enriched among patients with T3/4 tumors, N1/2 nodal status, and AJCC stage III disease, whereas no significant correlation was found with distant metastasis (M stage). These data suggest that clonal complexity constitutes an independent prognostic axis that complements, but is not captured by, the traditional TNM staging system. Collectively, these results provide a compelling molecular rationale for refined risk stratification, particularly among stage II–III CRC patients. Importantly, individuals with elevated ITH scores may benefit from intensified adjuvant therapeutic regimens or prioritized enrollment in clinical trials investigating immunotherapy–targeted therapy combinations to optimize long-term outcomes.

To develop a molecular prognostic model, we systematically evaluated 101 combinations of feature selection and modeling strategies based on 52 differentially expressed ITRGs significantly associated with OS in the context of ITH. The optimal model—a 9-gene signature constructed using Elastic Net feature selection (α = 0.5)—demonstrated strong predictive robustness across the TCGA training cohort and two independent GEO validation cohorts. Importantly, even after adjusting for age, clinical stage, and additional covariates, the resulting RS retained independent prognostic value, achieving time-dependent AUCs of 0.669, 0.664, and 0.645 for 1-, 3-, and 5-year survival predictions, respectively. Integration of the ITH score with key clinical parameters into a nomogram further enhanced its utility for long-term survival prediction and personalized clinical decision-making. Methodologically, our approach aligns with the ITRG model recently proposed by Chen et al. for cholangiocarcinoma, which similarly utilized DEGs and a suite of ten mainstream algorithms—including RSF, Lasso, Elastic Net, Ridge and CoxBoost—to generate 90 candidate models. Their optimal 12-gene Lasso model, selected on the basis of the cross-cohort mean C-index, showed outstanding prognostic performance in the TCGA cohort, with 2-, 3- and 4-year AUCs of 0.955, 0.950 and 1.000, respectively—far surpassing conventional clinical indicators such as age, tumor grade and TNM stage (29). Although the two studies differ in cancer type, biological context, and gene composition, both underscore the effectiveness of a systematic, multi-algorithm modeling strategy in improving model robustness and generalizability. Collectively, these findings highlight the broad applicability and independent prognostic relevance of ITRG signatures across diverse malignancies.

From an immunological perspective, our findings indicate that the ITRG-based risk stratification is not merely a prognostic tool, but rather delineates an ITH-driven axis of immune–stromal co-evolution. The high-ITH/high-risk subgroup as a whole exhibited a prototypical “inflammatory–stromal” tumor microenvironment: on the one hand, it was enriched for CAFs, tumor-associated macrophages and other stromal components; on the other hand, it was characterised by a relative depletion of effector and memory lymphocyte populations and by persistent activation yet functional dysregulation of multiple immune pathways. Thus, instead of representing a “hot tumor” state that is conducive to immune elimination, this pattern more closely resembles an immune-excluded microenvironment arising in the context of chronic inflammation, in which immune cells are sequestered at the tumor periphery and fail to exert effective cytotoxic activity. This phenotype is highly consistent with previously described mechanisms of CAF- and M2 macrophage–mediated immune escape, whereby these cell populations remodel the extracellular matrix and secrete TGF-β, IL-10 and other soluble mediators to create a collagen-dense, immunosuppressive niche that both impedes T-cell trafficking and dampens effector function (30–32). In keeping with this model, high-ITH tumors in our cohort displayed broad activation of inflammatory and immune-related pathways but dysregulation of the Th1/Th2/Treg axis and features of chronic immune stimulation coupled with functional exhaustion (33). At the transcriptomic level, this state was further reflected by widespread upregulation of immune checkpoint molecules, activation of immunometabolic suppressive pathways such as IDO1/2 and ADORA2A, and increased TIDE scores, consistent with our observation that high-risk tumors harboured higher TMB and a greater proportion of MSI-L/H cases. These findings indicate that even in the presence of relatively high TMB and MSI, high-ITH tumors tend to exhibit a pattern of chronically activated yet ultimately ineffective antitumor immunity, driven by coordinated immune–stromal remodeling. Similar ITH-associated features—namely, the coexistence of high clonal diversity, increased mutational burden and ineffective antitumor immunity—have been reported in gastric cancer and other solid tumors (34), supporting the notion that transcriptomic ITH scores do not simply capture random expression noise, but instead integrate clonal diversity, immune pressure and stromal reprogramming into a coherent immuno-ecological axis whose downstream consequence is immune escape rather than immune elimination.

Among the genes included in the prognostic model, Interleukin-20 Receptor Subunit β (IL20RB) exhibited the highest mean absolute SHAP value, identifying it as the most influential predictive feature within the ITRG signature. IL20RB is a shared subunit of the type II cytokine receptor family that heterodimerizes with IL20RA or IL22RA1 to activate downstream signaling pathways such as JAK1/STAT3 and NF-κB (35). It has established roles in mucosal barrier repair and chronic inflammation and has been implicated in promoting tumor progression in several solid malignancies. Previous studies have associated IL20RB overexpression with poor prognosis in pancreatic, lung and clear cell renal cell carcinomas (36–38); however, its role in CRC has remained largely unexplored. In this study, we report for the first time that IL20RB is significantly upregulated in CRC tissues and cell lines. Functional assays demonstrated that IL20RB silencing substantially suppressed CRC cell proliferation, migration and tumorigenic capacity *in vivo*, supporting its role as an oncogenic driver and validating its prominence within the prognostic model. Notably, correlation analyses suggested that IL20RB itself showed only modest associations with immune cell infiltration, indicating that it is more likely to confer proliferative and invasive advantages within high-ITH clones than to act as a sole determinant of the immunosuppressive microenvironment. Unlike traditional biomarker identification strategies based solely on differential gene expression, our integration of SHAP-based machine learning enabled precise prioritization and experimental validation of IL20RB as a key ITH-associated risk gene. This approach not only strengthens the biological interpretability of the model but also enhances its translational relevance in the context of CRC (39).

In summary, we developed and validated an RNA-seq–based ITH scoring system using the DEPTH2 algorithm and derived an ITRG-based risk signature that enables survival stratification and individualised risk assessment in CRC across multiple independent cohorts. The characteristic “inflammatory–stromal” tumor microenvironment observed in the high-ITH, high-risk subgroup, together with IL20RB-driven proliferative and invasive advantages at the clonal level, may jointly underlie the poor clinical outcomes in this population. These findings not only deepen the mechanistic understanding of how transcriptomic ITH relates to immune–stromal remodeling in CRC, but also provide a conceptual framework for rational combination strategies, including CAF and macrophage reprogramming, multi-target immune checkpoint blockade and IL20RB-directed interventions. Nevertheless, several limitations should be acknowledged. All datasets analysed were retrospective, the model was validated only in publicly available cohorts with heterogeneous platforms, and spatial information on intratumoral immune and stromal architecture was not available, which may limit generalisability and preclude definitive causal inferences. In addition, although IL20RB emerged as a key risk-associated gene and oncogenic driver, its precise immunomodulatory functions within the high-ITH microenvironment require further elucidation. Future studies leveraging multicenter prospective cohorts, spatial and single-cell omics and functional dissection of IL20RB signaling are warranted to confirm the clinical utility of the ITH score and to refine therapeutic strategies for high-ITH CRC subgroups.

## Data Availability

The original contributions presented in the study are included in the article/**Supplementary Material**. Further inquiries can be directed to the corresponding authors.
