# Supplementary material for: Intratumor heterogeneity score reveals immune landscape and survival stratification in colorectal cancer
Source: Front Immunol. 2026 Jan 5;16:1671148. doi: 10.3389/fimmu.2025.1671148 (PMC12813002; doi:10.3389/fimmu.2025.1671148)
Supplement: Supplementary file 1 [file Table1.docx]

Supplementary Material

**Supplementary **Figure 1. Distribution and prognostic performance of the ITRG-based RS across cohorts.** (A) Heatmaps showing expression patterns of the nine signature genes in the TCGA, GSE40967, and GSE87211 cohorts, ordered by increasing RS. (B) Distribution of RS in each cohort; the vertical dashed line indicates the median RS used to divide patients into high- and low-risk groups. (C) Scatter plots of survival status and survival time along the RS axis (orange dots, dead; purple dots, alive), illustrating the accumulation of death events in the high-risk group. (D) Time-dependent ROC curves for 1-, 3-, and 5-year OS in each cohort, with corresponding AUC values indicating the predictive accuracy of the ITRG-based signature.**


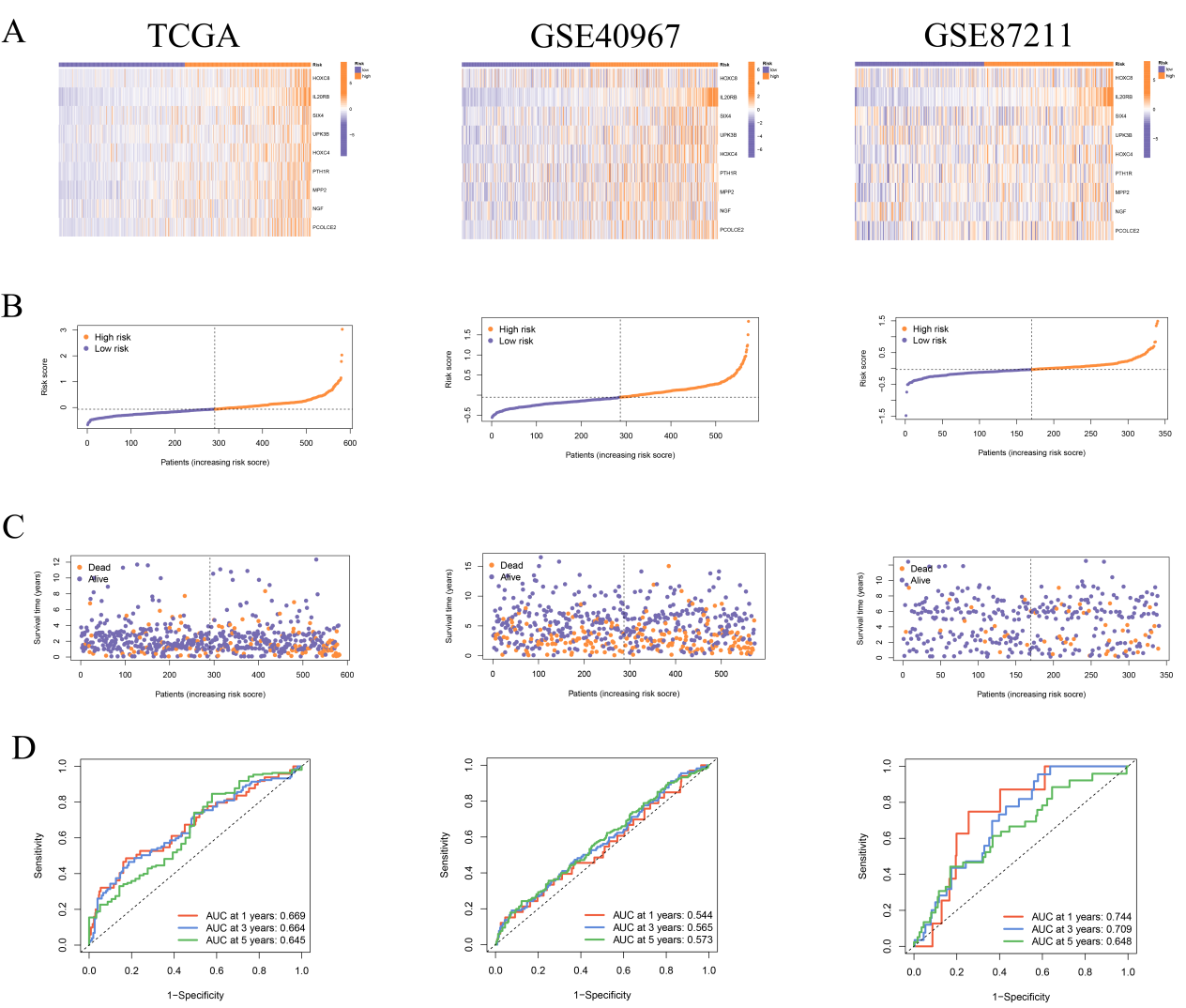


**Supplementary **Figure 2.** Genomic features associated with the ITRG-based RS in the TCGA CRC cohort.** (A) Boxplot comparing TMB between low- and high-risk groups (left) and scatter plot showing a positive correlation between RS and TMB, colored by risk group (right). (B) Distribution of the RS across microsatellite status subgroups (MSS, MSI-L, MSI-H). (C) Scatter plot showing a negative correlation between the RNAss and the RS, with marginal density plots indicating RNAss distributions in low- and high-risk groups. (D–E) Waterfall plots depicting the somatic mutation landscape of canonical CRC driver genes in the high-risk (D) and low-risk (E) groups, respectively. The upper bar indicates sample-level TMB, the right bar plots show mutation frequencies, and colors represent different mutation types.


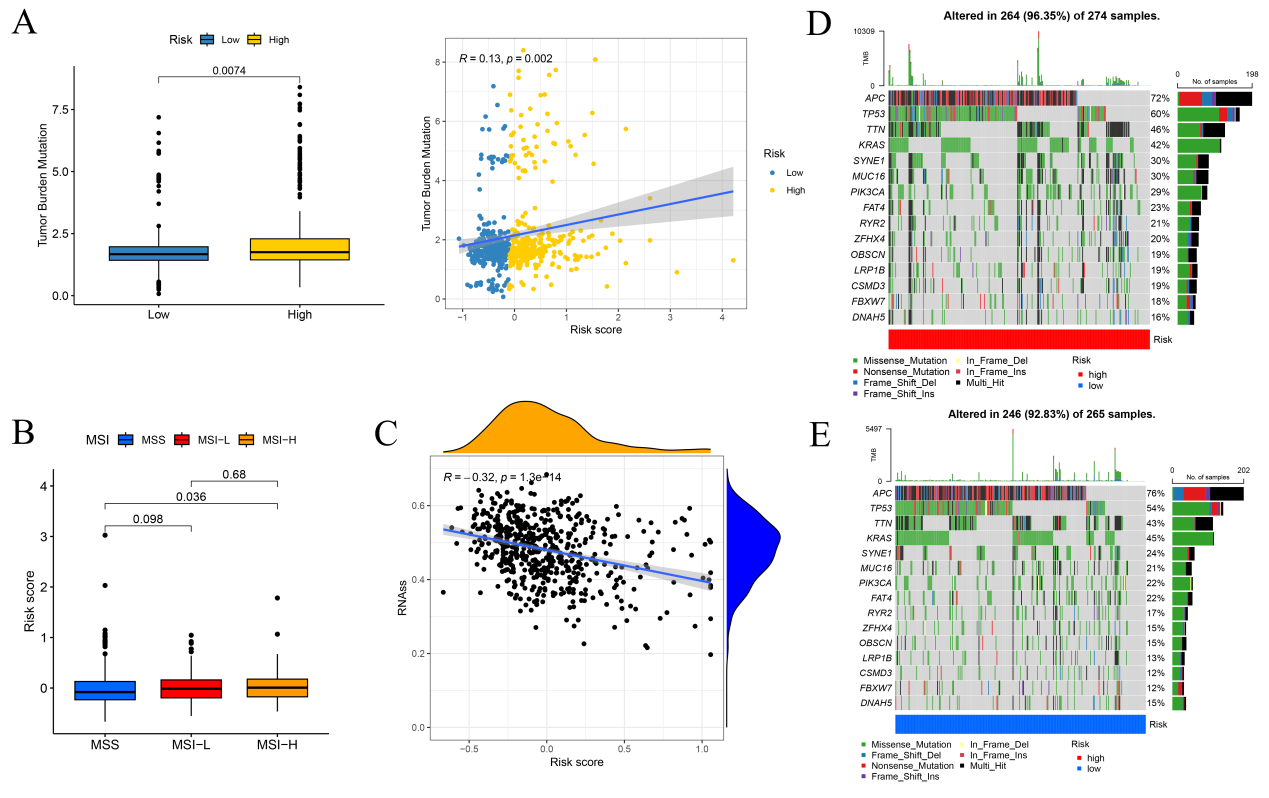


**Supplementary Table 1.** PCR primer sequences for IL20RB.

| **Gene symbol** | **Primer sequence** |
| --- | --- |
| ***IL20RB*** | **F:** GATGTGGAGCCCAGTGAT  **R:** CCTTCAGTGAGTGAGCACC |

**Supplementary Table S2.** Primary and secondary antibodies used in this study.

| Target (Antibody) | Supplier | Catalog No. | Working dilution |
| --- | --- | --- | --- |
| IL20RB | Proteintech | #20521-1-AP | 1:1000 |
| *β*-Actin | Proteintech | #66009-1-1g | 1:10000 |
